# Temperature Dependence of the Sensitivity of PVDF Pyroelectric Sensors to THz Radiation: Towards Cryogenic Applications

**DOI:** 10.3390/s24175808

**Published:** 2024-09-06

**Authors:** Artem N. Sinelnikov, Anatoly R. Melnikov, Yaroslav V. Getmanov, Darya A. Kolomeec, Evgeny V. Kalneus, Matvey V. Fedin, Sergey L. Veber

**Affiliations:** 1 Nesmeyanov Institute of Organoelement Compounds of the Russian Academy of Sciences, 28, Vavilova Str., Moscow 119334, Russia; sinelnikov.an@phystech.edu; 2 Moscow Center for Advanced Studies, 20, Kulakova Str., Moscow 123592, Russia; 3 International Tomography Center of the Siberian Branch of the Russian Academy of Sciences, 3a, Institutskaya Str., Novosibirsk 630090, Russia; mfedin@tomo.nsc.ru; 4 Novosibirsk State University, 1, Pirogova Str., Novosibirsk 630090, Russia; y_getmanov@mail.ru; 5 Budker Institute of Nuclear Physics of the Siberian Branch of the Russian Academy of Sciences, 11, Acad. Lavrentieva Ave., Novosibirsk 630090, Russia; darya.skorohod.88@gmail.com; 6 Novosibirsk State Technical University, 20, Karl Marx Ave., Novosibirsk 630073, Russia; 7 Voevodsky Institute of Chemical Kinetics and Combustion of the Siberian Branch of the Russian Academy of Sciences, 3, Institutskaya Str., Novosibirsk 630090, Russia; kalneus@kinetics.nsc.ru

**Keywords:** THz radiation, pyroelectric detector, polyvinylidene difluoride, pyroelectric coefficient, free-electron laser, cryogenic temperature

## Abstract

The application of terahertz (THz) science in industrial technology and scientific research requires efficient THz detectors. Such detectors should be able to operate under various external conditions and conform to existing geometric constraints in the required application. Pyroelectric THz detectors are among the best candidates. This is due to their versatility, outstanding performance, ease of fabrication, and robustness. In this paper, we propose a compact pyroelectric detector based on a bioriented poled polyvinylidene difluoride film coated with sputtered metal electrodes for in situ absorption measurement at cryogenic temperature. The detector design was optimized for the registration system of the electron paramagnetic resonance (EPR) endstation of the Novosibirsk Free Electron Laser facility. Measurements of the detector response to pulsed THz radiation at different temperatures and electrode materials showed that the response varies with both the temperature and the type of electrode material used. The maximum signal level corresponds to the temperature range of 10–40 K, in which the pyroelectric coefficient of the PVDF film also has a maximum value. Among the three coatings studied, namely indium tin oxide (ITO), Au, and Cu/Ni, the latter has the highest increase in sensitivity at low temperature. The possibility of using the detectors for in situ absorption measurement was exemplified using two typical molecular spin systems, which exhibited a transparency of 20–30% at 76.9 cm^−1^ and 5 K. Such measurements, carried out directly in the cryostat with the main recording system and sample fully configured, allow precise control of the THz radiation parameters at the EPR endstation.

## 1. Introduction

The development of efficient detectors of THz radiation is of paramount importance for various fields of daily life, such as safety technology, communications, biomedical applications, and non-invasive and non-destructive object detections [1,2,3,4,5,6,7]. At the moment, there are many THz radiation detectors operating on different physical principles. Some of them are relatively traditional ones, e.g., different kinds of bolometers, nanobolometers, pyroelectrics, and piezoelectrics. They transform a temperature change caused by THz radiation into an electrical signal via conductivity or polarization change [1]. There are also a considerable number of detectors based on more specific principles or materials. These include optically gated graphene [8], photomixers [9], single resonanttunneling diode oscillators [10], heterojunction/homojunction photoemission detectors [11], quantum dot detectors [12,13], various types of antenna detectors [14], Schottky diodes based on semiconducting single-walled nanotubes [15], and some other usually deep-cooled detectors that are used mainly for astronomical applications. Among others, Schottky barrier diodes are probably the best-known uncooled transducers in the THz frequency range, achieving noise equivalent power (NEP) up to 10^−12^ W·Hz^−1/2^ at 3.3 cm^−1^ (3000 μm; 0.1 THz) [16,17,18]. For polymer transducers, the lowest values of NEPs in the THz frequency range reported to date are of the order of 10^−8^–10^−7^ W·Hz^−1/2^ [19,20,21].

The response time of standard bolometric, pyroelectric, and piezoelectric detectors is usually relatively long (>100 ns), making them unsuitable for coherent detection. Instead, their broad spectral sensitivity, simple design, and decent NEP allow them to be used to measure the averaged radiant power. Crystalline pyroelectrics such as lead zirconate titanate (Pb[Zr_x_Ti_1−x_]O_3_ (0 ≤ x ≤ 1), PZT), ZnO, and lithium tantalite (LiTaO_3_) are widely used as motion sensors and as sensor arrays in commercial Fourier transform infrared (FTIR) spectrometers. LiTaO_3_ has a high pyroelectric coefficient of 176 μC·m^−2^·K^−1^ and a low dissipation factor, determined by a tanδ of 1.5·10^−4^ at 1 kHz [6,22]. PZT and its modifications have pyroelectric coefficients of up to 260 μC·m^−2^·K^−1^ that, together with their high relative permittivity of 380, allow them to be used in small energyharvesting systems [23,24,25]. The technological application of singlecrystal pyroelectrics can be further advanced by photolithography [26,27], which makes it possible to build a sensor together with a preamplifier on a single chip, e.g., Broadcom’s thinfilm pyroelectric infrared sensor [28]. Polymeric pyroelectric detectors based on, for example, polyvinylidene difluoride (PVDF) are in some cases more preferable than singlecrystal pyroelectrics due to their mechanical and chemical resistance, low cost, flexibility, and the possibility to be deposited on various types of substrates [29,30,31,32]. The high electric field resistivity of 1 MV·cm^−1^ makes it possible to polarize such polymer films by electric field without stretching directly on the already deposited films [10,11,12,13]. PVDF and its copolymers have a lower pyroelectric coefficient of 33 μC·m^−2^·K^−1^ compared to single crystals of LiTaO_3_ and PZT. At the same time, their relative permeability is about four times lower [33,34]. This reduces the capacitance of the sensitive area of the detector and increases the voltage response.

There are many radiation sources available in the THz frequency range, differing in mode of operation, available power, radiation coherence, wavelength tunability, etc. Among others, free-electron lasers (FELs) stand out for their ability to generate powerful, coherent, monochromatic THz radiation [35]. The Novosibirsk Free Electron Laser (NovoFEL) facility consists of three different FELs that utilize a recuperative scheme and are capable of generating radiation both in quasi-continuous and macropulse regimes [36]. The THz FEL of the facility operates in the 25–111 cm^−1^ (90–400 μm; 0.75–3.3 THz) spectral range, while the other two operate in the far-infrared and mid-infrared spectral ranges. The NovoFEL is a user-oriented facility. There are about a dozen specialized endstations devoted to fundamental research in various fields ranging from biology to THz optics [37]. The electron paramagnetic resonance (EPR) endstation is focused on the investigation of resonant and non-resonant interactions of THz radiation with the electron spins of different molecular compounds that mainly include coherent systems for the realization of electronic qubits and single-molecule magnets [38,39]. Experimental methods of EPR spectroscopy are typically based on the use of different microwave probeheads that are placed inside a helium cryostat to conduct experiments at low temperatures. The cryostat is located between the poles of the electromagnet. THz radiation is transmitted to the sample through a special multimodal hollow metal/dielectric waveguide with a length of about 60 cm and an outer diameter of 8 mm [40].

There are several cases when the ability to measure and control the absorption of the sample under study is of great importance in low-temperature experiments. Firstly, it allows the optical density of the sample to be controlled at the wavenumber and temperature used, providing an accurate parameter for optimizing the alignment of the optical system of the endstation. Secondly, for some systems, it allows to precisely tune the wavenumber of radiation to the energy splitting of levels within the spin system by searching for an absorption maximum in some spectral range. In this case, a resonant process is considered, where THz photons directly excite spin transitions or phonons in the molecular spin system under study. Usually, the resonance position can be determined with a high accuracy by FTIR or FD-FT THz-EPR spectroscopies [41,42]. To reduce the spin–spin interaction that limits the application of the EPR spectroscopy, a diamagnetic dilution is often utilized. The diamagnetic replacement is not always isostructural to the main paramagnet, which could lead to changes in the spin system parameters that determine the energy splitting [43]. The latter could even vary from synthesis to synthesis, since it is practically impossible to reproduce the degree of diamagnetic dilution exactly. In situ measurement of the transmittance of the sample pellet solves the problem described and allows the precise tuning of the THz radiation. Finally, low-temperature pyroelectricity is interesting even without the context described. This effect is believed to have simple features that are universal for a wide variety of solids at T near 0 K. There are comprehensive data on the temperature dependence of the pyroelectric coefficient of singlecrystal pyroelectrics, while polymeric analogues have been less studied [44,45,46,47,48,49].

Spatial limitations and the need to operate over a wide range of temperatures, from helium to room temperature, severely limit the feasibility of a number of the THz radiation detectors mentioned above. One of the most universal and robust of these are transducers based on PVDF polymer films. This flexible material allows the creation of specialized devices that can be adapted to existing geometric constraints. The NEP of these detectors is about 1–100 μW·Hz^−1/2^ in the THz range at room temperature and is low enough to measure the transmission through the sample of the rather powerful radiation of the THz FEL [50]. In this paper, we propose a design of the pyroelectric detector based on a bioriented poled PVDF film coated with sputtered electrodes that can be used for in situ measurement of THz radiation intensity at cryogenic temperatures. A custom printed circuit board (PCB) was used to create a device with the appropriate aperture and geometric dimensions and to connect it by a twisted pair to the preamplifier located outside the cryostat. The relative response of the detectors with indium tin oxide (ITO), Cu/Ni, and Au electrodes was obtained at a wavenumber of 76.9 cm^−1^ (130 μm; 2.31 THz) in the temperature range of 4–300 K, and possible factors influencing the response are discussed. The linearity of the detector response was checked at 5 K. The possible applications at the EPR endstation are discussed and illustrated by the in situ measurements of two typical molecular spin systems at 5 K.

## 2. Results and Discussion

### 2.1. General Design of the Experiment

Three types of pyroelectric detectors, differing in the conductive materials of the electrodes that cover the bioriented poled PVDF film on both sides and in optical properties in the THz frequency range [50], were investigated. These are (i) indium tin oxide (ITO), (ii) Cu/Ni, and (iii) Au. All PVDF films were manufactured by PolyK (PolyK Technologies, Philipsburg, PA, USA). The conductive electrode materials were sputtered on the PVDF films by the same company. The main characteristics of the films are given in Table 1. Hereinafter, pyroelectric detectors based on these PVDF films are named the same as electrodes.

The THz free-electron laser of the NovoFEL facility is used as a source of monochromatic THz radiation [37,52,53]. The available spectral range of 25–111 cm^−1^ (90–400 μm; 0.75–3.3 THz) allows the THz FEL to be used for the direct excitation of spin transitions when radiation is resonant with the energy splitting of levels within the spin system. The accuracy of the resonance can be determined by in situ measurement of the transmittance of a pellet of the compounds under investigation directly in the cryostat of the EPR spectrometer. The discussion continues in Section 2.5. A wavenumber of 76.9 cm^−1^ (130 μm; 2.31 THz) was used in all experiments. The choice of this wavenumber is due to the best long-term stability of the THz FEL operation at this frequency. The radiation spectrum is shown in Figure S1 of the Supporting Information. The NovoFEL facility was operated in the macropulse lasing mode that allows the use of pulsed radiation with a minimum duration determined by the Q-factor of the optical resonator of the THz FEL. Macropulses of 1 ms duration were used in all experiments at a repetition rate of 5 Hz. The typical rise and fall times of THz macropulses are about a few microseconds, so they can be neglected compared to the total pulse duration to simplify the subsequent analysis of the obtained data and to consider a pulse as a rectangular one. The repetition rate of 5 Hz allows complete cooling of the active element of the detector at all the cryostat temperatures used. The average THz radiation power was measured with a Gentec-EO UP19K-15S-VR detector (Gentec-EO, Quebec, QC, Canada) and was 0.5 W, 3.4 W, and 5.3 W for the ITO, Cu/Ni, and Au detector, respectively. The power was measured in front of the multimodal hollow metal/dielectric THz waveguide, so the actual power reaching the detector was at least two times lower [40]. The exact power level was adjusted to obtain approximately the same response level from the PVDF detectors at room temperature. This was achieved by means of placing several 1 mm thick polyethylene terephthalate (PET) films in the optical system of the EPR endstation.

**Figure 1:**
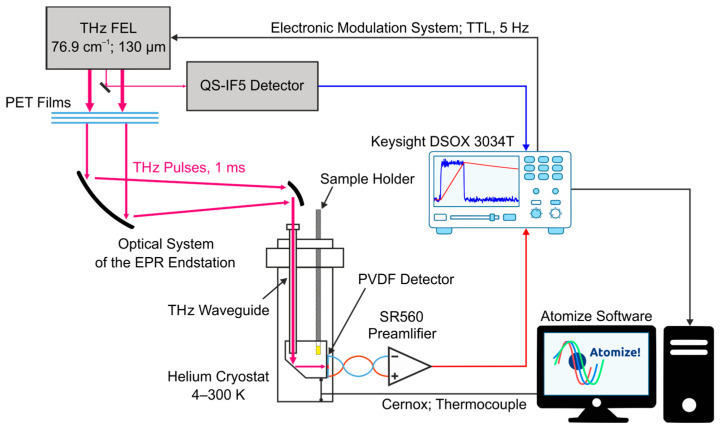
General scheme of the optical system of the EPR endstation, the detection system, and the location of the PVDF detector inside the helium cryostat.

To control the stability of the THz radiation from the NovoFEL over the duration of the experiment, the THz beam was split into two unequal parts. The first part of less than 5% of the total intensity was monitored by the QS-IF5 (Gentec-EO, Quebec, QC, Canada) pyrodetector. The main beam, after necessary attenuation, was directed by two mirrors of the optical system into the THz waveguide. The waveguide at one end was closed with a special cover with an optical window, which isolates the volume of the helium cryostat (Cryotrade Engineering, Moscow, Russia) from the atmosphere. The temperature of the cryostat was controlled by a LakeShore 335 temperature controller (Lake Shore Cryotronics, Westerville, OH, USA) equipped with a Cernox cryogenic temperature sensor and a type E thermocouple (both Lake Shore Cryotronics, Westerville, OH, USA), which provided temperature stability better than 0.05 K. All experiments were carried out in the temperature range of 4–300 K. The general scheme of the optical system of the EPR endstation, the detection system, and the location of the PVDF-based detector are shown in Figure 1.

**Figure 2:**
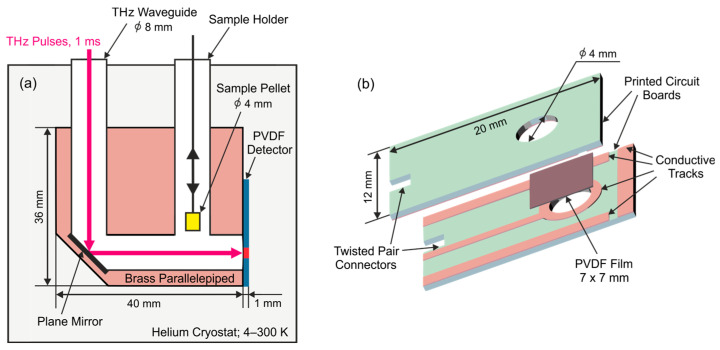
(**a**) The structure of the brass parallelepiped with the plane mirror and two functional axial holes for the THz waveguide and sample holder. The pyroelectric detector is fixed to the parallelepiped with four screws. (**b**) The general design of the sensing unit of the pyroelectric detector and its connection to the detection system.

The THz waveguide transfers radiation to a brass parallelepiped with a size of 20 × 40 × 36 mm, in which a mirror made of polished stainless-steel plate is mounted at an angle of 45°. The THz beam then reaches the pyroelectric detector, which is fixed on the opposite side of the parallelepiped with four screws. There is also space between the mirror and the detector for the sample to be inserted from the top of the cryostat into the second functional axial channel of the parallelepiped. The described configuration is shown in Figure 2a.

The pyroelectric detectors investigated consist of the changeable pyroelectric film attached to two custom PCBs by means of tensile forces, providing reliable electrical contact. The tracks on the sides of the PCBs were used to solder them together, eliminating any movement of the film. To expose the film to THz radiation, there are two circular holes of 4 mm diameter in the circuit boards. Electrical contact is made between the circumference of the holes and the PVDF film electrodes. The general design of the detector is shown in Figure 2b. A photograph of the assembled detectors is shown in Figure S2 of the Supporting Information.

### 2.2. Approach to Measuring Detector Response

**Figure 3:**
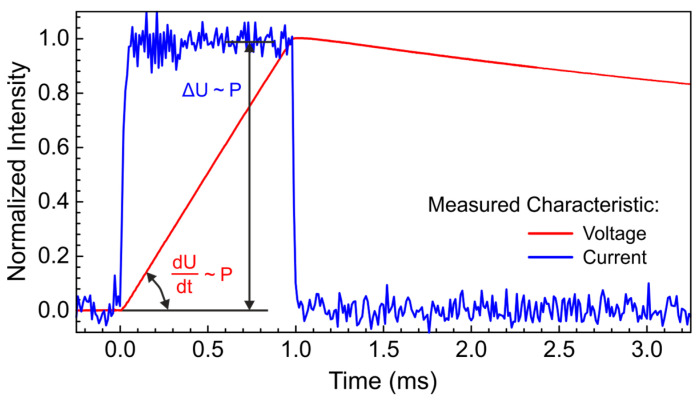
Examples of the response of the pyroelectric detectors measured by two different approaches, namely by a transimpedance amplifier measuring a constant current, blue solid line, QS-IF5 pyrodetector, and by registering the voltage on the sides of the sensing film and further amplifying by the high-impedance amplifier with a gain of 100, red solid line, ITO pyrodetector.

The pyroelectric film covered with two electrodes to collect the polarization charge forms a capacitor. When exposed to THz radiation, the pyroelectric material absorbs some of it, causing it to heat up. At the beginning of the heating process, the temperature change is small, which means that the heat transfer to the environment is negligible, and the heating rate is constant. The charge (Δq) on the sides of the pyroelectric film is determined by the temperature change (ΔT) and the pyroelectric coefficient (p) as follows: Δq = pΔT. At the same time Δq = CΔU, where C is the capacitance of the two electrodes, and ΔU is a voltage across them. The time derivative gives the current as I = CdU/dt = pdT/dt. It follows from this equation that there are two approaches to measuring the response of a pyroelectric detector. The first is to measure a heat-induced current. This is typically done using a transimpedance amplifier [21]. The output of the amplifier delivers a voltage that is proportional to the intensity of the THz radiation. For example, the average THz radiation power of the NovoFEL is measured at the EPR endstation in parallel to the experiments (see also Section 2.1) using the QS-IF5 pyrodetector equipped with the transimpedance amplifier. Although this is a fairly straightforward method, the measurement of constant current at constant THz radiation power is affected by the bias current of the amplifier, which could reduce the accuracy. The noise caused by capacitive coupling is also high when the amplifier is far away from the sensor. In our case, to perform measurements at cryogenic temperatures, the amplifier has to be located outside the cryostat, which creates additional noise sources. Because of that, we chose the second method that is based on registering the voltage on the sides of the sensing element. The voltage from the detector is further amplified by an SR560 high-impedance amplifier (Stanford Research System, Sunnyvale, CA, USA) with a gain of 100. The amplifier is connected to the pyrodetector via a twisted pair cable. The impedance of the SR560 is so high (>100 MΩ) that the RC time of the system is about 20 times longer than the duration of the experiment. A 50 Ω output of the SR560 amplifier is further connected to a 350 MHz Keysight DSOX 3034T oscilloscope (Keysight Technologies, Santa Rosa, CA, USA) that is used to record the voltage curve. The arbitrary waveform generator of the same oscilloscope is used to trigger the electronic modulation system of the NovoFEL, which generates the THz macropulses. To obtain the THz radiation intensity from the measured voltage, the first derivative should be taken. Examples of the raw experimental data measured by both methods are shown in Figure 3. The measurements were fully automated using the open-source software Atomize 0.1.0 [54].

### 2.3. Factors Affecting Detector Response

There are several factors that influence the response of the detector when the temperature is changed. This is not only a change in the pyroelectric coefficient [47] but also a change in the heat capacity of the sensor. As the temperature decreases, the pyroelectric film heats up faster during the THz pulse due to its lower heat capacity at these temperatures [55]. Another factor is the change in the dielectric constant of the pyroelectric material [56], as this affects the capacitance of the sensor across which the voltage is measured. To best of our knowledge, there is no detailed information on the dielectric constant of PVDF at low temperatures. Therefore, the exact degree of its influence on the recorded signals cannot be estimated directly. Finally, the reflectivity of the pyroelectric film electrodes and the efficiency of the THz beam transport system, including a part of the multimodal THz waveguide (see Figure 1 and Figure 2) also changes with temperature. It is quite difficult to disentangle the individual contributions described, so we looked at the overall response of the sensor in the temperature range of 4–300 K.

During the measurements, the microphone effect was also observed, which gradually increased the noise level, especially at low temperatures. This is probably caused by perturbations in the helium flow through the cryostat, as it increases significantly when approaching a temperature of about 4 K. We addressed this issue by extending the measurement time to more thoroughly average the raw data. If measurement time is critical, implementing a local deflecting screen and some damper constructions may be required to minimize helium flow disturbances near the pyroelectric film.

### 2.4. Temperature Dependence of the Detector Response

**Figure 4:**
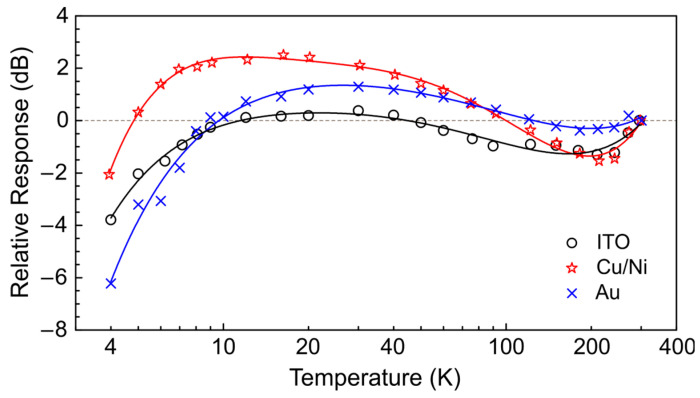
Temperature dependence of the response of the pyroelectric detectors with different sputtered electrodes. Symbols show: “○”—ITO; “☆”—Cu/Ni; “x”—Au. The response is normalized to room temperature. Solid lines are guides for the eye.

The obtained dependencies of the relative responses of the detectors with different sensitive elements on the cryostat temperature are shown in Figure 4. In all cases, a similar behavior of the detector response was observed that can be roughly divided into three temperature ranges: (i) 300–200 K, (ii) 200–20 K, and (iii) 20–4 K. In the first range, a slight decrease in sensitivity was observed. At temperatures below 200 K, the detector sensitivity increases that is followed by an abrupt drop at temperatures below 20 K. The behavior obtained is mainly determined by the temperature dependence of the pyroelectric coefficient of the PVDF film [56]. The latter is known to have a maximum value at temperatures of about 14–60 K, depending on the exact type of sensing material [44]. At lower temperatures, it usually decreases, but the quantitative characteristics depend on the material and its crystal structure if singlecrystal detectors are considered [47]. Although, in general, the temperature dependence can be explained by a change in the magnitude of the pyroelectric coefficient, its quantitative behavior remains different for different electrode coatings. These more subtle distinctions can be attributed to the heat capacity of the sensor as a whole and changes in the reflectivity of the electrode material. The latter cannot explain the entire dependence obtained because the reflectivity of metal films usually increases at low temperature as the conductivity of metals increases [57]. This reduces the amount of THz radiation absorbed by the pyroelectric, while the opposite trend was observed in the experiment. However, the discrepancy in the measured temperature dependence for the detectors with different electrode materials can be due to changes in the reflectivity of the latter.

Another important characteristic of the detector is its voltage response. It determines the absolute value of the response of the detector to the applied radiation. In the context of the proposed in situ measurement of sample transmittance, this provides an estimate of the minimum possible transmittance that can be detected under typical experimental conditions. Taking into account the upper limit estimate of the THz power applied to the detectors (see Section 2.1) and the absolute level of the recorded signals, the voltage response at the applied amplifier gain and room temperature for the ITO, Cu/Ni, and Au detectors are as follows: 1.1 V/W, 0.1 V/W, and 0.1 V/W. This means that a transmittance level of 1% can be routinely measured if 1 W THz power is applied to the sample, as this corresponds to a signal level of 1–10 mV at the applied amplifier gain. This power level is achievable for the THz FEL of the NovoFEL facility. The higher voltage response of the ITO detector is consistent with the lower NEP of this detector, which was previously measured at 66.7 cm^−1^ (150 μm; 2.0 THz) [50] and was found to be determined by the higher radiation absorption of the ITO sputtered PVDF film in the THz frequency range. As for the temporal characteristics of the investigated detectors, the typical response times of the PVDF-based detectors of similar design were found to be of the order of microseconds at room temperature [50]. The temperature dependence of the response time was not investigated in detail in the present work, but we did not observe any significant changes in it during low-temperature measurements.

**Figure 5:**
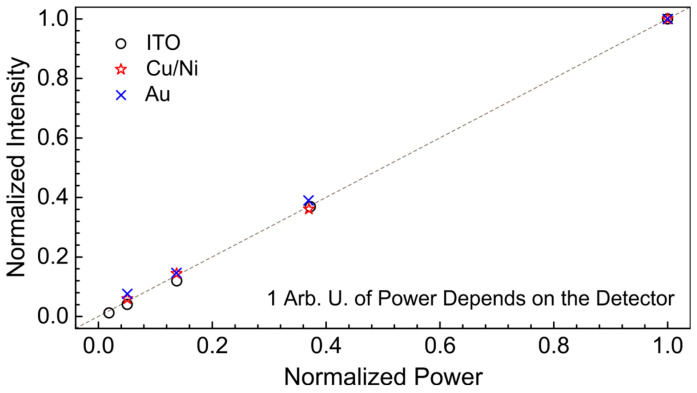
Linearity of the detector responses measured at 5 K. Symbols show: “○”—ITO; “☆”—Cu/Ni; “x”—Au; the dashed line is a guide for the eye; 1 arb. u. of power and intensity depends on the detector used and is in the range of 1–3 W and 100–300 mV, respectively.

Figure 5 demonstrates the linearity of the detector responses measured at 5 K. As mentioned above, the different level of the applied THz power was achieved by placing several 1 mm thick PET films in the optical system of the EPR endstation. At a wavenumber of 76.9 cm^−1^ (130 μm; 2.31 THz), one such PET film attenuated radiation about 2.7 times. According to Figure 5, all the detectors studied exhibit linear behavior even at the rather high applied THz power of about 1 W per sensitive area with a diameter of 4 mm.

### 2.5. Application at the EPR Endstation

The sensitivity obtained at cryogenic temperatures allows the detectors to be used routinely for in situ measurement of the intensities and shapes of THz pulses passing through the samples under investigation. At the EPR endstation, the latter are usually different molecular spin systems that exhibit phonon absorption lines or energy splitting of spin levels in the THz frequency range. In cases where the exact resonance position is known, such miniature detectors allow control of the optical density of the sample at the used wavenumber and temperatures. Given the low frequency of the radiation used, the simple room temperature measurements using standard FTIR spectroscopy are not feasible. A further difficulty is that the sample holders used at the EPR endstation are optimized for pellets with diameters of 4, 3, or 1.5 mm, as determined by the size restrictions of registration apparatus, for instance, by a microwave probehead. Such diameters are substantially smaller than the typical pellet size for a commercial FTIR spectrometer. This significantly reduces the available aperture and, consequently, the sensitivity of the spectrometer.

As for the energy of zero-field splitting in high-spin systems, this value was found to be sensitive to the degree of dilution by a diamagnetic analogue that is not always isostructural. For example, a progressive dilution of paramagnetic Co(II) by diamagnetic Zn(II) in [Co_x_Zn_1−x_(piv)_2_(2-NH_2_-Py)_2_]—where x is in the range of 1–0, piv is pivalate, and 2-NH_2_-Py is 2-aminopyridine—beyond 50% reveals a structural change [43]. The change is accompanied by a modification of the zero-field splitting, determining the resonance of the spin-system. This means that even for the systems fully characterized by other techniques, there is always a chance that a newly synthesized compound will have a different resonance position. It is quite difficult to repeat the degree of diamagnetic dilution exactly, as this process is a kind of statistical one. By scanning the frequency of the THz FEL and directly measuring the transmittance of the sample, the exact resonance position can be found for the sample used.

As a proof of concept, we checked that the intensity of the radiation passed through a typical sample pellet is enough for reliable signal registration. The Au detector, which showed the lowest sensitivity in the THz range, was utilized. Two compounds, namely [VO(TPP)], where TPP is 5,10,15,20-tetraphenylporphyrin, and [CoTp_2_], where Tp is bis[tris(pyrazolyl)borate], were chosen as model examples of molecular spin systems. Their chemical structure is given in Figure S3 of the Supporting Information. They were finely ground, mixed with ultra-high-molecular-weight polyethylene powder (Micro Powders, Tarrytown, NY, USA), and pressed into 4 mm pellets approximately 2 mm thick. The pellets contain 2.7 mg of [VO(TPP)] and 1.5 mg of [CoTp_2_]. Both molecules have no strong absorption bands at a wavenumber of 76.9 cm^−1^ (130 μm; 2.31 THz). A transparency of 20–30% with a signal-to-noise ratio ≥ 50 was obtained at 5 K in a oneminute measurement, indicating the feasibility of the proposed approach for fine-tuning THz frequency using in situ measurements. The experimental data are shown in Figures S4 and S5 of the Supporting Information.

## 3. Conclusions

In this paper, the properties of three pyroelectric polymer detectors were studied in order to utilize them at cryogenic temperatures. The proposed design of the detector has the geometric dimensions optimized for the helium cryostat of the EPR endstation. The custom printed circuit boards together with voltage registration on the sides of the sensing element provide a reliable electrical contact and a good signal-to-noise ratio, even if the sensing element is located 60 cm away from the high-impedance preamplifier. Performed measurement of the detector response to pulsed THz radiation of 76.9 cm^−1^ (130 μm; 2.31 THz) as a function of temperature reveals that the response is mainly determined by the temperature dependence of the pyroelectric coefficient of the PVDF film. The sensitivity has a maximum in the range of 10–40 K and decreases by about 1.5–2 times at 4 K. The quantitative behavior depends on the type of the sputtered electrodes, showing that the heat capacity of the sensor and changes in the reflectivity of the electrodes also play a role in temperature dependence. Of the three coatings studied, Cu/Ni has the highest increase in sensitivity in the 10–40 K temperature range, which is at the level of +2 dB relative to room temperature, and simultaneously has the lowest sensitivity decrease at helium temperatures, which only reaches the level of –2 dB. All the detectors linearly respond to THz radiation of varying power up to 1 W per sensitive area, with a diameter of 4 mm at helium temperatures. Finally, the performance obtained allows the absorption of the sample pellet to be measured in situ at low temperatures. Transparency of the order of a few percent with a signal-to-noise ratio of about 100 can be measured in a few minutes of acquisition, as was exemplified by two typical molecular spin systems at 5 K. Such in situ measurements enable ultimate control of the THz beam characteristics at cryogenic temperatures in a confined space, such as the EPR probehead at the EPR endstation of the NovoFEL.

## Figures and Tables

**Table 1:** Several main characteristics of the investigated pyroelectric detectors.

Detector Name		ITO	Cu/Ni	Au
Sensitive element	Material	PVDF ^1^	PVDF ^1^	PVDF ^1^
	Thickness (μm)	28	28	12
	Diameter (mm)	4	4	4
Electrodes	Material	ITO ^2^	Cu/Ni ^2^	Au ^2^
	Thickness (nm)	2500 ^3^	70/10	100

^1^ Polymer film of polyvinylidene fluoride; ^2^ sputtered metal electrodes; ^3^ calculated based on ITO sheet resistance of 300 Ω/sq, specified by the manufacturer, using a resistivity of 7.5 × 10^−4^ Ω·cm [51]. All the presented characteristics except the diameter of the sensitive element are provided by the manufacturer.

## Data Availability

The raw experimental data are available from the corresponding authors upon reasonable request.

## Supplementary Materials

The following supporting information can be downloaded at https://www.mdpi.com/article/10.3390/s24175808/s1: Figure S1, radiation spectrum used for the performance study of the detectors at 76.9 cm^−1^ (130 μm; 2.3 THz); Figure S2, photographs of the assembled detectors based on PVDF film covered on both sides by three different types of electrodes—indium tin oxide (ITO), Cu/Ni, and Au, as indicated on them. The electrodes were made by metal sputtering. The geometrical dimensions are indicated on the Au detector; Figure S3, structures of the compounds used in this work—[VO(TPP)], where TPP is 5,10,15,20-tetraphenylporphyrin, and [CoTp_2_], where Tp is bis[tris(pyrazolyl)borate]; Figure S4, response of the Au detector to THz radiation with a wavenumber of 76.9 cm^−1^ (130 μm; 2.31 THz) measured with (solid dark green line) and without (solid navy line) sample pellet. The pellet contains 1.5 mg of [CoTp_2_]; Figure S5, response of the Au detector to THz radiation with a wavenumber of 76.9 cm^−1^ (130 μm; 2.31 THz) measured with (solid dark green line) and without (solid navy line) sample pellet. The pellet contains 2.7 mg of [VO(TPP)].
