# Supplementary material for: Temperature Dependence of the Sensitivity of PVDF Pyroelectric Sensors to THz Radiation: Towards Cryogenic Applications
Source: Sensors (Basel). 2024 Sep 6;24(17):5808. doi: 10.3390/s24175808 (PMC11398077; doi:10.3390/s24175808)
Supplement: Supplementary file 1 [file sensors-24-05808-s001.zip › sensors-3179129-supplementary.pdf]

# Supporting Information

# Temperature Dependence of the Sensitivity of PVDF Pyroelectric Sensors to THz Radiation: Towards Cryogenic Applications

**Artem N. Sinelnikov [1,2,3], Anatoly R. Melnikov [3,*], Yaroslav V. Getmanov [4,5,6], Darya A. Kolomeec [5], Evgeny V. Kalneus [7], Matvey V. Fedin [3,4] and Sergey L. Veber [3,4,*]**

[1] Nesmeyanov Institute of Organoelement Compounds of the Russian Academy of Sciences, 28, Vavilova Str., Moscow 119334, Russia; sinelnikov.an@phystech.edu
[2] Moscow Center for Advanced Studies, 20, Kulakova Str., Moscow 123592, Russia
[3] International Tomography Center of the Siberian Branch of the Russian Academy of Sciences, 3a, Institutskaya Str., Novosibirsk 630090, Russia; mfedin@tomo.nsc.ru
[4] Novosibirsk State University, Department of Physics, 1, Pirogova Str., Novosibirsk 630090, Russia; y _getmanov@mail.ru
[5] Budker Institute of Nuclear Physics of the Siberian Branch of the Russian Academy of Sciences, 11, Acad. Lavrentieva Ave., Novosibirsk 630090, Russia; darya.skorohod.88@gmail.com
[6] Novosibirsk State Technical University, Electrophysical Installations and Accelerators Department, 20, Karl Marx Ave., Novosibirsk 630073, Russia
[7] Voevodsky Institute of Chemical Kinetics and Combustion of the Siberian Branch of the Russian Academy of Sciences, 3, Institutskaya Str., Novosibirsk 630090, Russia; kalneus@kinetics.nsc.ru
* Correspondence: anatoly.melnikov@tomo.nsc.ru (A.R.M.); sergey.veber@tomo.nsc.ru (S.L.V.)

## Contents

S1. Radiation Spectrum S2
S2. Photograph of the Detectors S3
S3. Structures of the Compounds Used S4
S4. Additional Figures S5

## S1. Radiation Spectrum

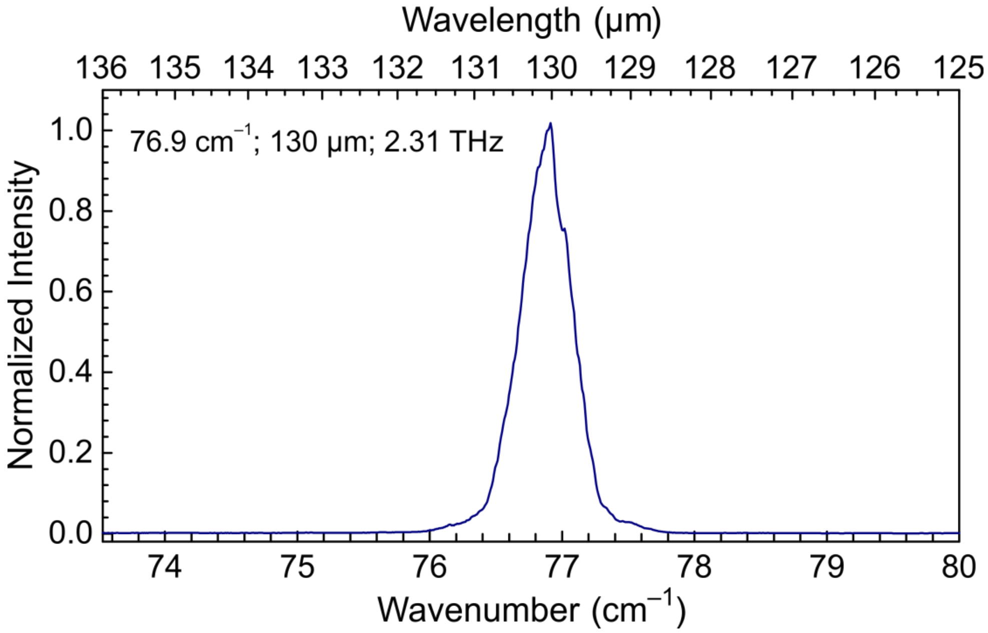


**Figure S1.** Radiation spectrum used for the performance study of the detectors at 76.9 cm$^{-1}$ (130 μm; 2.3 THz).

## S2. Photograph of the Detectors

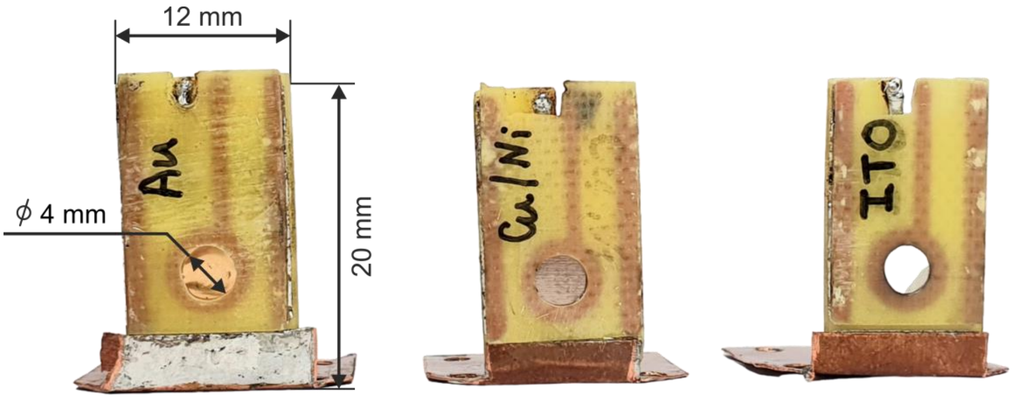


**Figure S2.** Photographs of the assembled detectors based on PVDF film covered on both sides by three different types of electrodes: indium tin oxide (ITO), Cu/Ni, and Au as indicated on them. The electrodes were made by metal sputtering. The geometrical dimensions are indicated on the Au detector.

## S3. Structures of the Compounds Used

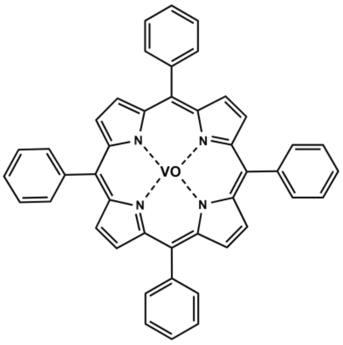

[VO(TPP)]

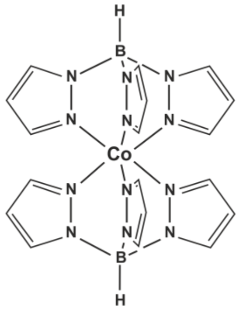

[$CoTp_2$]

**Figure S3.** Structures of the compounds used in this work: [VO(TPP)], where TPP is 5,10,15,20-tetraphenylporphyrin; [$CoTp_2$], where Tp is bis[tris(pyrazolyl)borate].

## S4. Additional Figures

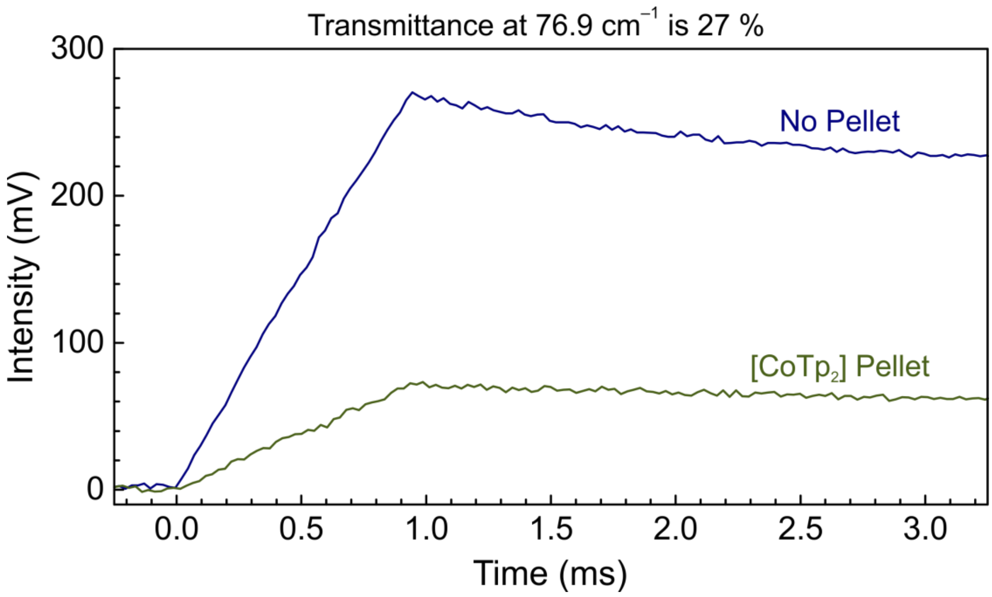


**Figure S4.** Response of the Au detector to THz radiation with a wavenumber of 76.9 cm$^{-1}$ (130 μm; 2.31 THz) measured with (solid dark green line) and without (solid navy line) sample pellet. The pellet contains 1.5 mg of [CoTp$_2$].

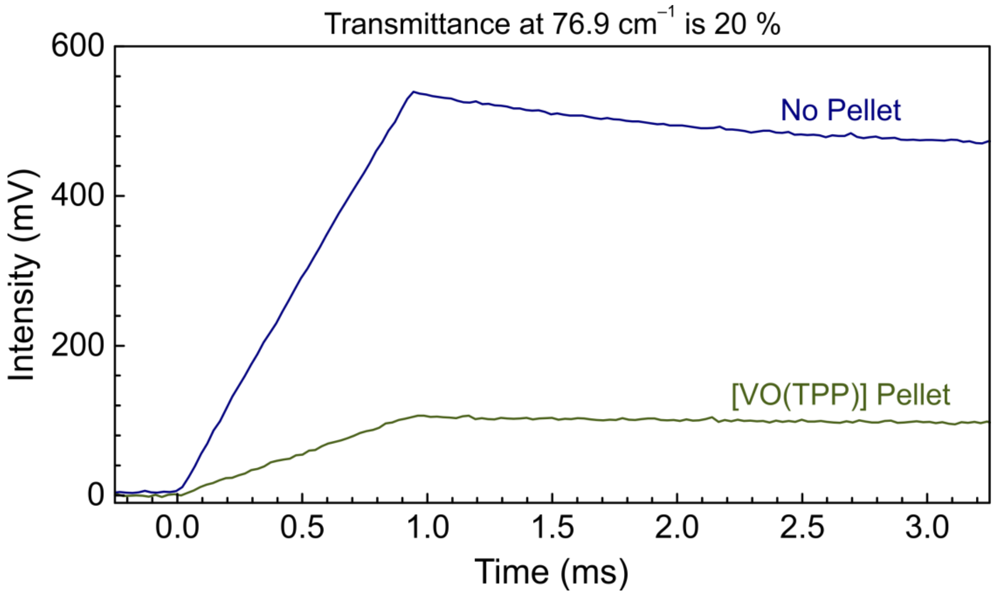


**Figure S5.** Response of the Au detector to THz radiation with a wavenumber of 76.9 cm$^{-1}$ (130 μm; 2.31 THz) measured with (solid dark green line) and without (solid navy line) sample pellet. The pellet contains 2.7 mg of [VO(TPP)].
